# Identification, Determination and Transdermal Behavior Characterization of Nine Components in WenTong HuoXue Cream by UPLC-MS/MS

**DOI:** 10.3390/ph19060805

**Published:** 2026-05-22

**Authors:** Xinran Zhang, Xiaodan Qiu, Xiaolong Kang, Guangzhi Shan, Chenghui He

**Affiliations:** 1 School of Pharmacy, Xinjiang Medical University, No. 567 Shangde North Road, Urumqi 830017, China; 2 Institute of Medicinal Biotechnology, Peking Union Medical College, Chinese Academy of Medical Sciences, No. 1 Tian Tan Xi Li, Beijing 100050, China; 3 Xinjiang Uygur Autonomous Region Institute of Traditional Chinese Medicine, No. 116 Huanghe Road, Urumqi 830002, China; 4 Xinjiang Key Laboratory of Uyghur Medical Research, Xinjiang Institute of Materia Medica, Urumqi 830004, China

**Keywords:** UPLC-MS/MS, WenTong HuoXue Cream, chemical constituents, quality control, transdermal behavior

## Abstract

**Background/Objectives:** WenTong HuoXue Cream (WTHXC) plays a significant role in the treatment of diabetic peripheral neuropathy (DPN). However, the material basis and quality control methods for this formulation remain largely unexplored. **Methods:** In this study, UPLC-HRMS/MS combined with standard reference substances was employed to comprehensively identify and confirm the chemical constituents of WTHXC. Furthermore, a rapid and sensitive UPLC-MS/MS method based on multiple reaction monitoring (MRM) mode was developed and validated for the simultaneous quantification of the marker components. **Results:** Nine compounds were unambiguous characterized, including Di-hydrocapsaicin (DHC), Oxypeucedanin hydrate (OPH), Imperatorin (IMP), Isoimperatorin (IIMP), Xanthotoxin (XAN), Hydroxysafflor yellow A (HSYA), Chlorogenic acid (CGA), Ferulic Acid (FA) and Ligustilide (LIG). The results of method validation denotes that all the analytes showed good linearity between concentration and peak area in the tested ranges, with correlation coefficients (r) not less than 0.9990. The relative standard deviation (RSD) of precision was in the range of 0.57–7.11%. The accuracy of the method, verified by recovery experiments at three concentration levels, ranged from 96.51% to 101.04% for all analytes. Transdermal behavior determination results demonstrate that OPH, HYSA, CGA, FA and LIG exhibited favorable skin permeability and may serve as the key active components of WTHXC. **Conclusions:** This study elucidates the material basis of WTHXC, providing a scientific foundation for the development of quality control methods and facilitating its broader clinical application.

## 1. Introduction

WenTong HuoXue Cream (WTHXC), an in-hospital preparation in Xinjiang District, has been utilized in clinical practice for nearly two decades [1]. This preparation is formulated with seven traditional Chinese medicinal ingredients: Chuanxiong Rhizoma, Angelicae Dahuricae Radix, Cinnamomi Ramulus, Capsici Fructus, Carthami Flos, Asari Radix et Rhizoma, and Pheretima [2]. Their corresponding original medicinal plants are *Ligusticum chuanxiong* Hort., *Angelica dahurica* (Fisch. ex Hoffm.) Benth. et Hook. f., *Cinnamomum cassia* Presl, *Capsicum annuum* L., *Carthamus tinctorius* L., *Asarum heterotropoides* Fr. Schmidt var. *mandshuricum* (Maxim.) Kitag., and *Pheretima aspergillum* (E. Perrier), respectively. In addition, the formulation contains excipients including stearic acid, glyceryl monostearate, lanolin, liquid paraffin and laurocapram.

WTHXC is primarily indicated for the treatment of Bi syndrome (impediment pattern) associated with Xiaoke Bing (wasting-thirst disease, analogous to diabetes mellitus), which corresponds to diabetic peripheral neuropathy (DPN). It is scientifically formulated based on the traditional Chinese medicine “monarch, minister, assistant and guide” theory, with each medicinal herb possessing well-defined medicinal characteristics: Chuanxiong Rhizoma as the principal drug regulates qi and blood to target DPN’s peripheral nerve inflammation and blood stasis syndrome; Carthami Flos as the ministerial drug enhances blood circulation to relieve DPN-related microcirculatory disturbance, peripheral nerve inflammation and nerve cell ischemia-hypoxia; the five adjuvant herbs (Angelicae Dahuricae Radix, Cinnamomi Ramulus, Asari Radix et Rhizoma, Pheretima, Capsici Fructus) act synergistically: Cinnamomi Ramulus warms meridians for cold congealing syndrome and peripheral inflammation; Asari Radix et Rhizoma penetrates skin to relieve neuroinflammation-induced neuropathic pain; Pheretima unblocks meridians, improves microcirculation and reduces peripheral nerve inflammatory infiltration in blood stasis syndrome; Capsici Fructus exerts analgesic/anti-inflammatory effects and enhances transdermal absorption of other components [3,4]. Modern pharmacological studies have revealed that key active constituents in the formulation exhibit a range of bioactivities. These include phthalides and phenolic acids from principal drug Chuanxiong Rhizoma [5,6,7], flavonoids from ministerial drug Carthami Flos [8], as well as coumarins from the adjuvant herb Angelicae Dahuricae Radix [9,10,11]. These components possess anti-inflammatory, analgesic, and antioxidant properties, and have been shown to improve microcirculation and alleviate or repair peripheral nerve damage [12,13,14,15,16]. Additionally, previous studies suggest that the topical efficacy of WTHXC may be associated with its involvement in anti-inflammatory responses, antioxidative stress mechanisms, and the modulation of redox imbalance [17,18,19,20].

Currently, research on WTHXC has primarily focused on its therapeutic mechanisms. However, systematic investigations into its material basis and quality control methods remain scarce. As a result, the safety, effectiveness, and quality controllability of this preparation remain unassured. This situation also poses limitations to the research on the pharmacodynamic substance basis of this drug and its in-depth clinical application. Mass spectrometry plays a pivotal role in investigating the material basis of pharmaceuticals [21]. Initial identification of components was achieved by matching MS1 and MS2 data against spectral databases. This was followed by rigorous confirmation using reference standards, where retention times were utilized to further validate the assignments [22]. Consequently, this integrated approach significantly enhanced the accuracy of component identification [23,24,25,26]. Among all quality control methods, Ultra Performance Liquid Chromatography-Tandem Mass Spectrometry (UPLC-MS/MS) offers distinct advantages such as high specificity, sensitivity, and analytical throughput [27,28]. This method allows for the rapid quantification of multiple components while enabling the sensitive detection and accurate quantification of low-abundance compounds [29,30]. Currently, UPLC-MS/MS is a powerful and reliable analytical technique widely applied in the qualitative and quantitative analysis of traditional Chinese medicine (TCM) components, featuring high separation efficiency, excellent sensitivity and strong specificity for accurate identification of multiple active ingredients and quality control of TCM [31,32,33,34,35].

Based on these features, this study firstly identified and confirmed nine components of WTHXC. Then, a UPLC-MS/MS method was developed and validated for the determination of the main components in WTHXC. In addition, the transdermal behavior of the above components was characterized in order to explore its mechanism of action. The aim is to provide an objective and comprehensive reference for the quality control of this preparation and the research on its material basis.

## 2. Results and Discussion

### 2.1. Identification and Confirmation of the Active Components in WTHXC Using UPLC-Orbitrap-MS/MS

**Figure 1:**
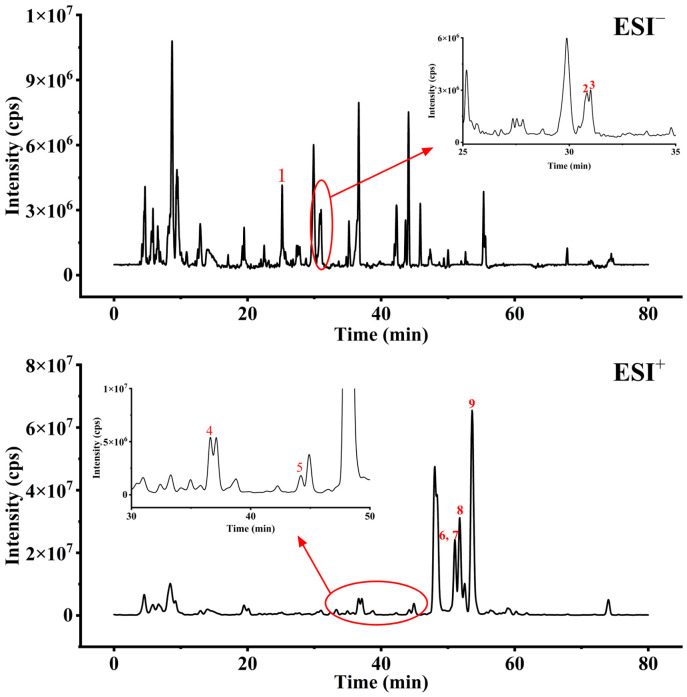
Total Ion Chromatogram of the WTHXC. Numbers 1–9 indicate CGA, HSYA, FA, OPH, XAN, DHC, IMP, LIG, and IIMP, respectively.

**Figure 2:**
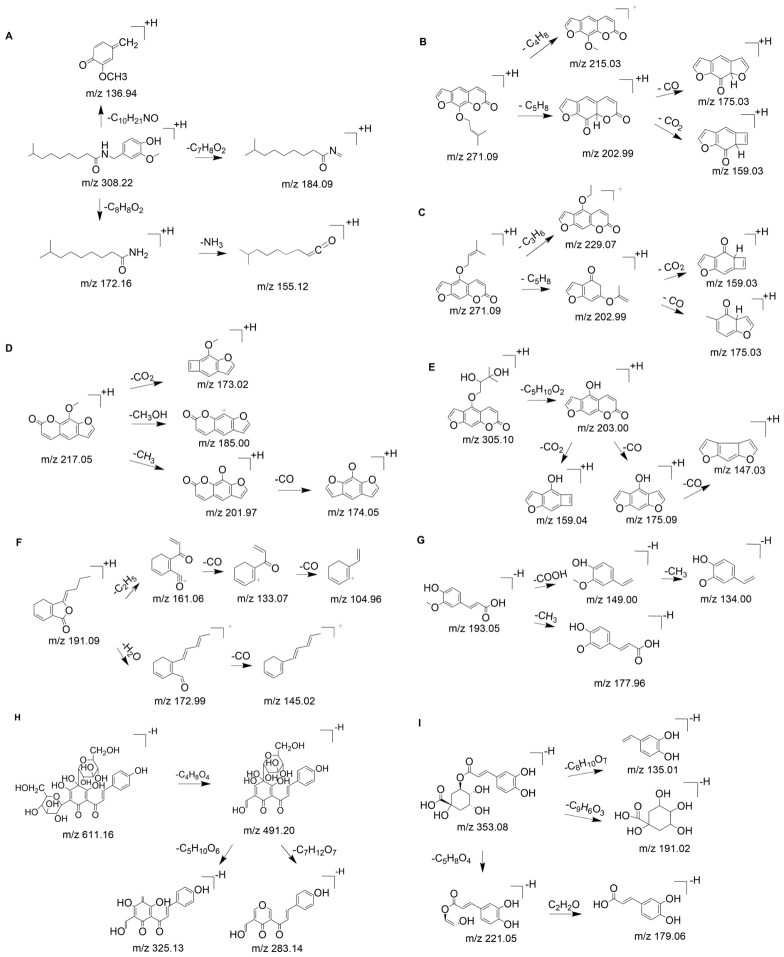
Fragmentation pathways of the nine compounds in WTHXC. (**A**) Proposed fragmentation pathway of DHC. (**B**) Proposed fragmentation pathway of IMP. (**C**) Proposed fragmentation pathway of IIMP. (**D**) Proposed fragmentation pathway of XAN. (**E**) Proposed fragmentation pathway of OPH. (**F**) Proposed fragmentation pathway of LIG. (**G**) Proposed fragmentation pathway of FA. (**H**) Proposed fragmentation pathway of HSYA. (**I**) Proposed fragmentation pathway of CGA.

To characterize the chemical composition of WTHXC, a systematic analysis was performed using UPLC-Orbitrap-MS/MS with full-scan mode coupled to data-dependent acquisition. The accurate precursor masses (MS1) and characteristic fragment ion spectra (MS2) of all detectable constituents in the samples were acquired. Raw mass spectral data were then imported and processed using Compound Discoverer 3.3.1.111. Candidate compounds were screened by matching accurate mass values against natural product databases. Preliminarily, 99 potential chemical constituents (including isomers) were identified in WTHXC by matching MS1 accurate masses (mass error ≤ 10 ppm) and MS2 fragment profiles with database entries Further validation by comparison of MS1, MS2 spectral data and retention times (retention time deviation ≤ 0.2 min) with authentic references ultimately enabled the unambiguous confirmation of nine constituents (Table 1, Figure 1 and Figure 2). The above components include four coumarins (IMP, IIMP, OPH, XAN), one flavonoid (HSYA), one phthalide (LIG), two phenylpropanoids (CGA and FA), and one alkaloid (DHC).

The nine selected marker compounds collectively represent the seven medicinal materials contained in WTHXC. HSYA is the characteristic flavonoid of Carthami Flos and acts as its primary quality control marker [36]. DHC is a representative capsaicinoid alkaloid derived from Capsici Fructus [37]. LIG is the characteristic phthalide constituent of Chuanxiong Rhizoma [38]. IMP, IIMP and OPH are the major bioactive components of Angelicae Dahuricae Radix. Among them, IMP and IIMP occur at relatively high levels and possess stable chemical properties, which makes them widely adopted marker compounds for traditional Chinese medicinal preparations containing this herb [39]. CGA and FA are phenylpropanoids ubiquitously distributed in Cinnamomi Ramulus and Chuanxiong Rhizoma. Owing to their prominent antibacterial and anti-inflammatory activities, both compounds are frequently employed as quality markers; in particular, FA has been extensively applied in the standardization of traditional Chinese medicines [40]. In conclusion, the selection of these nine compounds as analytical markers for WTHXC is well justified by their diverse chemical structures, potential bioactivities closely associated with the indications of WTHXC, as well as good commercial accessibility [41]. The strategy of adopting the above nine components as quality markers provides a scientifically reliable basis for the quality evaluation of this formulation.

### 2.2. Determination of Nine Components Using UPLC-TQS-MS/MS

#### 2.2.1. Development and Optimization of Sample Preparation Method

**Figure 3:**
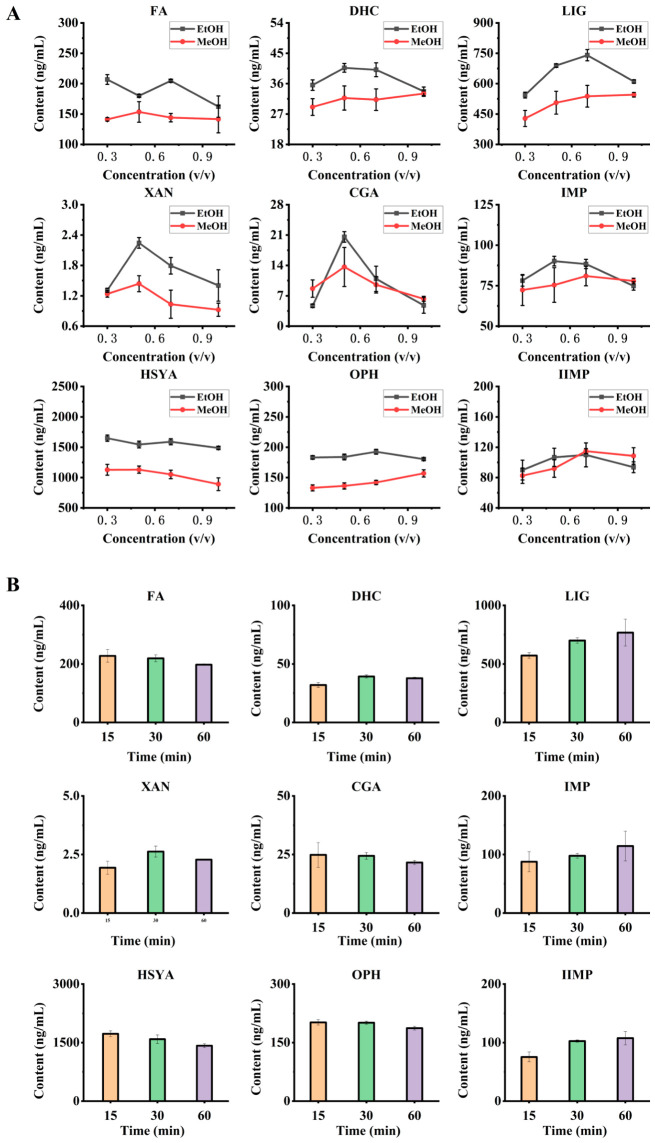
Optimization of extraction solvents (**A**) and durations (**B**).

WTHXC comprises multiple medicinal herbs containing diverse compounds with varying physicochemical properties. To establish an efficient extraction protocol, this study evaluated the effects of different extraction solvents (methanol, 30% methanol, 50% methanol, 70% methanol, ethanol, 30% ethanol, 50% ethanol, and 70% ethanol) and extraction durations (15, 30, and 60 min of ultrasonication) on the recovery of target analytes. As illustrated in Figure 3A, ethanol exhibits a better extraction performance than methanol. In terms of extractant proportion, different extractants impose varying effects on different analytes. Overall, 70% ethanol delivers the most favorable extraction efficiency for the nine components. The non-monotonic trend of FA extraction efficiency with the change in extractant proportion may be attributed to the mismatched polarity of FA with the extraction solvent and the encapsulation effect induced by demulsification of the cream matrix.

Regarding the optimization of ultrasonic extraction time, an extraction duration of 30 min can comprehensively balance extraction throughput and extraction efficiency (Figure 3B). The decline in the extraction efficiency of certain components with the extension of ultrasonic time can be explained by the following potential causes. WTHXC is an oil-in-water cream matrix; excessive ultrasonication induces oil–water phase redistribution, causing active components to remain trapped in the oil phase. In addition, the prolonged thermal effect may also cause slight degradation of heat-sensitive constituents. Finally, the use of 70% ethanol combined with 30 min of ultrasonication was selected for the extraction [42,43].

#### 2.2.2. Optimization of Chromatographic and Mass Spectrometric Conditions

**Figure 4:**
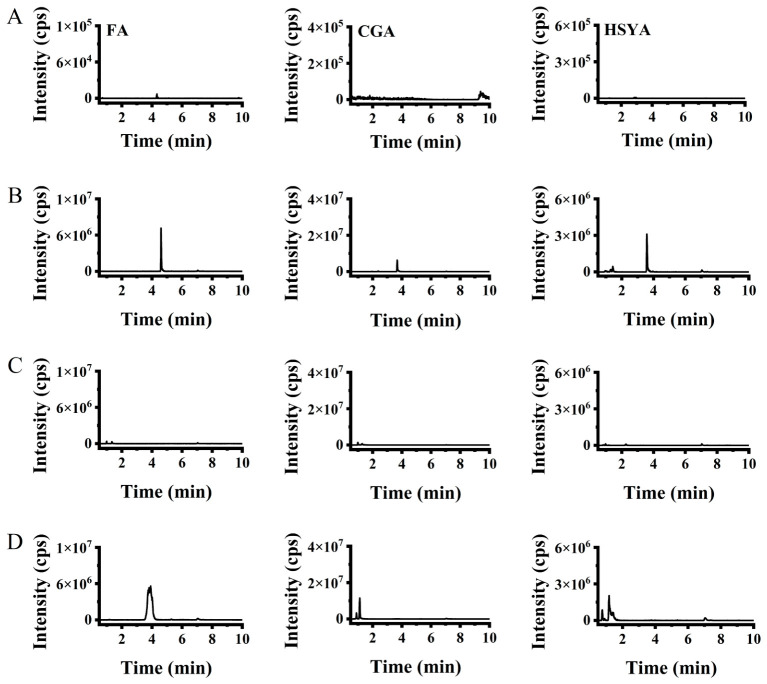
Comparison of chromatograms of WTHXC on BEH C18 column-0.1% formic acid aqueous solution-acetonitrile (**A**), HSS T3 column-0.1% formic acid aqueous solution-acetonitrile (**B**), HSS T3 column-10 mM ammonium acetate aqueous solution-acetonitrile (**C**), and HSS T3 column-water-acetonitrile (**D**).

To achieve efficient separation of all constituents in WTHXC, the present study optimized the chromatographic column and mobile phase conditions. Given that the other six components (DHC, IMP, IIMP, XAN, OPH and LIG) exhibited favorable responses in the positive electrospray ionization (ESI^+^) mode, this section primarily focuses on the chromatographic and mass spectrometric behaviors of the remaining three analytes (FA, CGA and HSYA) in the negative electrospray ionization (ESI^−^) mode. Overall, ACQUITY UPLC HSS T3 presented remarkable superiorities over BEH C18 (Figure 4A,B). Under identical mobile phase conditions, CGA and HSYA exhibited severe peak tailing and weak response on the BEH C18 column, while the HSS T3 column yielded sharp and symmetric peak shapes for all analytes with significantly enhanced response intensity. This enhancement is attributed to the unique bonding technology of the HSS T3 stationary phase, which effectively shields the activity of silanol groups and strengthens the retention of polar compounds. Accordingly, the HSS T3 column was selected for subsequent mobile phase optimization.

Three mobile phase systems were evaluated: 0.1% formic acid aqueous solution-acetonitrile, 10 mM ammonium acetate aqueous solution-acetonitrile, and pure water-acetonitrile (Figure 4C,D). Results showed that the pure water or ammonium acetate system caused serious peak tailing or weak response, whereas the addition of formic acid notably improved peak symmetry. Specifically, the 0.1% formic acid system adjusted the mobile phase to strong acidity, which efficiently suppressed the dissociation of acidic groups and enabled analytes to exist as molecules species without dissociation, thus achieving the optimal peak shape and retention. Nevertheless, in ESI^−^ mode, formic acid added to the mobile phase benefits peak shape optimization but suppresses analyte ionization, which inevitably decreases the mass spectral response of target analytes (Figure 4B,D). Taking both separation efficiency and mass spectrometry compatibility into comprehensive consideration, the HSS T3 column coupled with the 0.1% formic acid aqueous solution-acetonitrile mobile phase system was finally determined as the optimal chromatographic condition for this study [44]. The elution mode was optimized in this study as well. The nine components in WTHXC exhibit significant differences in polarity, including both highly polar and weakly polar constituents. Under such conditions, isocratic elution fails to achieve the separation and determination of all components within a reasonable analysis time. In contrast, gradient elution dynamically adjusts the elution strength of the mobile phase, enabling the quantification of all target analytes within a relatively short analysis duration. Based on the above considerations, gradient elution was adopted in this study to achieve satisfactory retention of highly polar components and effective elution of weakly polar components.

#### 2.2.3. Method Validation

The validation of the UPLC-MS/MS method for the analysis of nine components in WTHXC were carried out by estimating specificity, linearity, precision, repeatability, stability, robustness and accuracy according to the ICH guideline 02 (R1) [45].

##### Specificity

**Figure 5:**
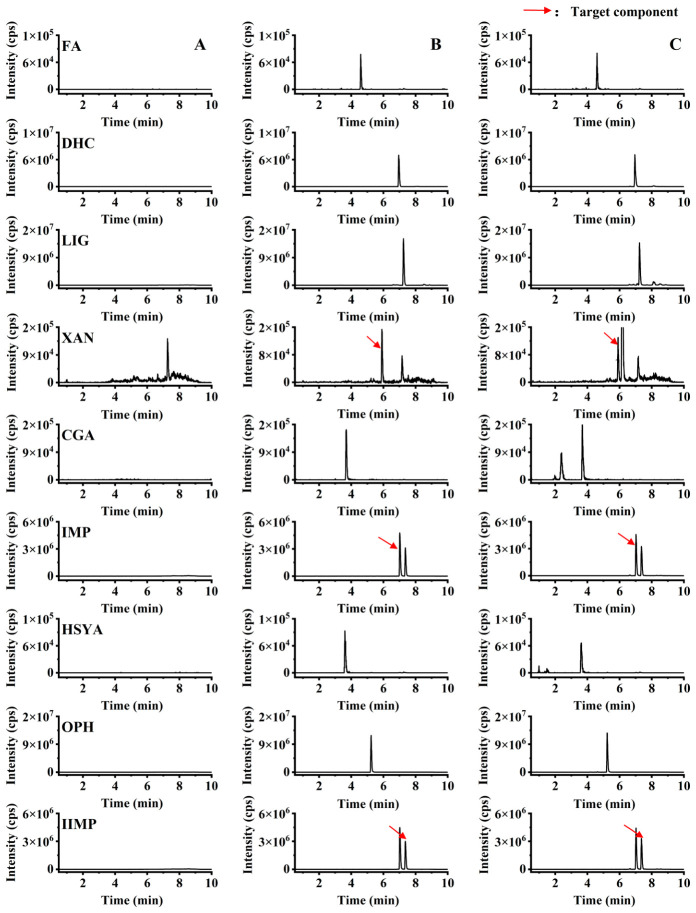
The MRM chromatograms of blank (**A**), reference standards (**B**) and WTHXC sample solution (**C**). Red arrows indicate the target components.

This study systematically evaluated the specificity of the analytical method by comparing the blank solvent, the mixed reference standard, and the test sample (Figure 5). The results showed that the blank solvent had no response at the retention time of the target analytes, eliminating the interference from the solvent and the mobile phase. This chromatographic method exhibited excellent specificity, effectively separating the analyte from potential interfering substances, and meeting the quality control requirements for the compounds.

##### Linearity

A series of working standard solutions at different concentrations were prepared by diluting an appropriate amount of the reference substance solution with 70% ethanol. These solutions were then injected and analyzed under the specified chromatographic and mass spectrometric conditions. A regression curve was constructed by plotting the peak area (Y) against the concentration of the reference substance (X). As shown in Table 2, the results demonstrated good linearity for all components within their respective concentration ranges, with all correlation coefficients (*r*) greater than 0.999.

##### Precision

The precision of the instrument was evaluated by repeatedly injecting a fixed volume of the reference standard solution under the specified analytical conditions. The relative standard deviations (RSDs) of the peak areas for the analyzed compounds—DHC, IMP, IIMP, OPH, XAN, HSYA, CGA, FA, and LIG—were 1.25%, 0.81%, 0.86%, 1.45%, 5.62%, 7.11%, 2.25%, 6.39%, and 0.57%, respectively (Table 2). These results demonstrate that the instrument precision is satisfactory.

##### Repeatability

The repeatability of the method was assessed by preparing six independent test solutions from accurately weighed samples and analyzing them under the specified conditions. The RSDs of the peak areas for the nine analyzed compounds—DHC, IMP, IIMP, OPH, XAN, HSYA, CGA, FA, and LIG—were 3.58%, 3.74%, 3.81%, 4.23%, 2.36%, 9.15%, 5.71%, 8.64%, and 4.29%, respectively (Table 2). These results confirm that the method exhibits good repeatability.

##### Stability

The stability of the test solution was assessed by analyzing aliquots at 0, 2, 4, 6, 12, and 24 h. The RSDs of the peak areas for the nine analytes—DHC, IMP, IIMP, OPH, XAN, HSYA, CGA, FA, and LIG—were 5.45%, 4.52%, 4.34%, 4.76%, 5.01%, 4.16%, 4.18%, 3.73%, and 5.13%, respectively (Table 2). These results demonstrate that all components remained stable within the 24 h testing period.

##### Accuracy

The recovery rate was determined by accurately transferring a fixed volume of the test sample stock solution and spiking it with known amounts of the reference standard solution at three concentration levels (80%, 100%, and 120% of the target). The resulting solutions were then prepared and analyzed according to the established method. The recovery rates were calculated, and the results are presented in Table 2. The results showed that at the low, medium and high concentration levels, the average recovery rates of the nine target analytes ranged from 96.51% to 101.04%, and the RSD was all less than 3.4%. These data indicate that the method has high accuracy, meeting the requirements for accurate quantification of these nine components in the samples.

##### Robustness

Robustness evaluation was conducted by systematically varying method parameters and the effects of column temperature (30 ± 2 °C), flow rate (0.3 ± 0.05 mL/min), and initial mobile phase composition (90 ± 5%) on the determination of WTHXC were investigated. Under different experimental conditions, the RSDs of the contents of DHC, IMP, IIMP, OPH, XAN, HSYA, CGA, FA, and LIG were determined as 2.15%, 1.14%, 8.79%, 6.91%, 6.77%, 7.11%, 8.10%, 2.30%, and 0.91%, respectively (Table 2). The results indicate that slight variations in the aforementioned experimental conditions exert no significant influence on the content determination results.

#### 2.2.4. Quantification of Nine Components in Six Batches of WTHXC

Different batches of WTHXC were dissolved in 70% ethanol, followed by ultrasonication, cooling and dilution. After centrifugation, the supernatant was collected and determined for the contents of nine components using the established UPLC-TQS-MS/MS method (Table 3). The content distribution showed that LIG exhibited the highest content across all batches (4.9308–6.7791 mg·g^−1^), establishing it as the core active substance in the formulation. In contrast, XAN showed the lowest content (0.0112–0.0322 mg·g^−1^), indicating its relatively minor proportion in the prescription (approximately 0.0026%). Analysis of batch-to-batch variability revealed that IIMP displayed the most significant variation in content (0.3784–1.0206 mg·g^−1^, with a relative fluctuation exceeding 2.7-fold), which may be attributed to differences in raw material batches, extraction processes, or formulation stability. All batches were characterized by LIG and HSYA as the predominant components. The observed fluctuations in certain components suggest the need for further optimization of raw material quality control and preparation processes to enhance batch-to-batch consistency of the formulation.

### 2.3. In Vitro Percutaneous Permeation Characterization Using UPLC-TQS-MS/MS

Wentong Huoxue Cream exerts its therapeutic effects mainly via topical application on the skin surface. Therefore, characterizing the transdermal behavior of its various components is conducive to enhancing the understanding and analysis of the material basis and mechanism of action of this cream. The intradermal retention amount reflects the component’s ability to accumulate in the skin and therefore we firstly employed the UPLC-MS/MS method to determine the retention behavior of the above nine components in the skin. According to the data in Table 4, it can be seen that the intradermal retention amounts of each component show significant differences. HYSA exhibits the highest intradermal retention, far exceeding other components. Next in order are LIG, FA and OPH, which constitute the high retention. This indicates that LIG, FA, OPH and HYSA have a strong affinity for the skin tissue and can effectively penetrate the stratum corneum barrier and accumulate within the skin. This high retention characteristic may be related to their molecular structure, lipophilicity, and interaction with skin. For applications aimed at treating local skin disorders (such as anti-inflammatory and antioxidant), these high retention components have the potential to become ideal candidate drugs, as they can maintain an effective concentration at the target site for a long time. In contrast, XAN and DHC exhibited extremely low intradermal retention levels. This may suggest that these components have difficulty penetrating the keratinized layer to exert some effects.

The content of the penetration fluid is a key indicator for evaluating whether the components can penetrate the entire skin barrier and enter the circulatory system to exert systemic effects. According to Table 4, among all the components, HSYA has the highest content in penetration fluid, significantly higher than all the other components, indicating that HSYA has excellent transdermal penetration ability and can efficiently penetrate the skin barrier. This may be attributed to its moderate molecular weight, good water-lipid partition coefficient, or other characteristics that facilitate transmembrane transport. Therefore, HSYA is the most promising active substance among these components for being developed as an active substance for systemic therapeutic effects through the transdermal delivery system. Followed closely are OPH and CGA, which also show certain penetration capabilities. DHC and IIMP have a penetration volume of approximately 0, while IMP and XAN, have extremely low penetration volumes, basically considered unable to effectively penetrate the skin, indicating that the skin barrier has almost completely blocked these components (Table 4).

Overall, the permeability values of all determined constituents in this study are consistent with those reported in the literature. For instance, previous studies have demonstrated that HSYA can permeate excised skin from W/O microemulsions, greatly facilitating its transdermal delivery [46]. The steady-state permeation flux of FA in gel formulations has been documented to range from 5.27 to 12.04 μg·cm^−2^·h^−1^ [47]. DHC and IMP exhibit favorable skin permeability when incorporated into transdermal patches and liposomal preparations, respectively [48]. CGA is inherently characterized by low transdermal permeability, thus, microemulsion carriers are required to achieve efficient skin delivery [49]. As the major lipophilic constituent of the volatile oil from Chuanxiong Rhizoma, LIG also displays skin permeation-enhancing effects [50].

By comparing the in vitro retention in skin and in vitro transdermal permeation values of the same components, we can gain a deeper understanding of their transdermal transport behavior patterns. Generally, components with higher concentrations in the permeate also show higher intradermal retention, such as OPH and HYSA, showing the potential of both local retention and systemic permeation. Notably, the retention of CGA in the skin was lower than that of FA and LIG, whereas its content in the permeate was considerably higher than those of FA and LIG. This may be related to differences in the physicochemical properties, skin tissue binding affinity, and metabolic stability of the three aforementioned compounds. Most components exhibit a much higher intradermal retention amount than the permeating fluid content. This is a typical transdermal absorption pattern, where the skin acts as a “drug reservoir”, with a large amount of components being retained, and only a small portion being able to eventually pass through. For FA and LIG, although they can enter the skin, they are extremely difficult to pass through, indicating their local action on the skin.

**Figure 6:**
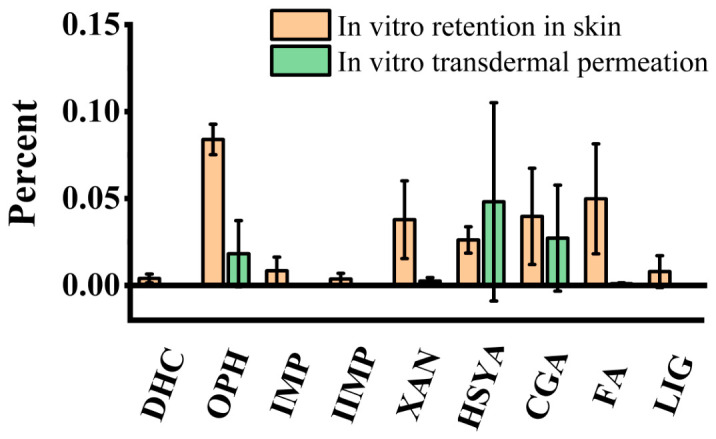
Comparison of the in vitro transdermal content and intradermal retention content of the 9 components in WTHXC.

We also investigated the ratios of the contents of nine components in the skin and permeate to their contents in the preparation, to characterize whether the relatively high levels of each substance in the permeate and skin were attributable to their high contents in the formulation. As shown in Figure 6, the ratios of the permeated amounts of OPH, HYSA, CGA, and FA to their original contents in the preparation were relatively high, indicating that their favorable transdermal behavior was mainly due to their good intrinsic permeability. Notably, although XAN showed a relatively low concentration in the skin, it exhibited a high penetration ratio, indicating favorable permeability. Its low concentration in the skin was mainly attributed to its low content in the formulation. LIG was the most abundant component in the formulation, yet its concentrations in both the penetration fluid and skin were lower than those of HYSA, suggesting that its permeability was inferior to HYSA. Similarly, despite their high contents in the formulation, IMP and IIMP still maintained low concentrations in the skin and penetration fluid, demonstrating that the poor penetration behavior of these compounds was determined by their inherent properties. Another notable phenomenon is that the standard deviations of some compounds are relatively large, especially for HSYA (4.47% ± 135.04%), CGA (2.72% ± 112.26%), and OPH (1.83% ± 104.03%). This high variability may be attributed to the following factors: Firstly, the inherent biological differences in skin samples are the main source of variation in in vitro permeation experiments. The thickness of the stratum corneum, lipid composition, and hair follicle density of different skin samples all affect the permeability of the drug; Secondly, the inherent error of low-concentration detection. Due to the cumulative permeation amount of some samples approaching the quantitative lower limit of the analytical method, the slight fluctuations of the instrument may lead to the amplification of the relative standard deviation.

## 3. Materials and Methods

### 3.1. Chemicals and Reagents

WTHXC (New Drug Preparation Approval No. Z20052604, Batch No. 20240804, 20250701, 20250702, 20250703, 20251022, 20251023) was manufactured by the the Affiliated Traditional Chinese Medicine Hospital of Xinjiang Medical University in Urumqi, China. Dihydrocapsaicin (DHC, purity ≥ 98%, Batch No. LD30Z43) and xanthotoxin (XAN, purity ≥ 98%, Batch No. BD20056-1g) were purchased from J&K Scientific Co., Ltd. (Beijing, China). Imperatorin (IMP, purity ≥ 98%, Batch No. JB256320), isoimperatorin (IIMP, purity ≥ 98%, Batch No. JB256320) and hydroxysafflor yellow A (HSYA, purity ≥ 98%, Batch No. JB258648) were obtained from Shanghai Yuanye Bio-Technology Co., Ltd. (Shanghai, China). Oxypeucedanin hydrate (OPH, purity ≥ 98%, Batch No. C17599512) and FA (FA, purity ≥ 98%, Batch No. C17487265) were sourced from Shanghai Macklin Biochemical Co., Ltd. (Shanghai, China). Chlorogenic acid (CGA, purity ≥ 98%, Batch No. KB363783) and ligustilide (LIG, purity ≥ 98%, Batch No. 24241028003) were purchased from Beijing Solarbio Science & Technology Co., Ltd. (Beijing, China). Ethanol (MS grade, Batch No. LC50Z05) was obtained from J&K Scientific Co., Ltd. (Beijing, China). Acetonitrile (MS grade, Batch No. F25P47201), methanol (MS grade, Batch No. LF50Z32) and formic acid (MS grade, Batch No. 226099) were purchased from Thermo Fisher Scientific Co., Ltd. (Shanghai, China).

### 3.2. Experimental Conditions

#### 3.2.1. UPLC-Orbitrap-MS/MS

The analytical procedure employed a U3000 high-performance liquid chromatography system (Thermo Fisher Scientific, Waltham, MA, USA) coupled with a quadrupole/electrostatic field orbitrap high-resolution mass spectrometer (Thermo Fisher Scientific, Waltham, MA, USA) for the detection of WTHXC. Chromatographic separation was achieved on an OSAKA SODA CAPCELL PAK C18 AQ S3 column (Osaka, Japan, 4.6 × 250 mm, 3 μm) maintained at a temperature of 30 °C. The mobile phase consisted of eluent A (0.1% formic acid in water, *v*/*v*) and eluent B (acetonitrile). The optimized gradient elution program was set as follows: 0–5 min, 5% B; 5–60 min, 5–90% B; 60–70 min, 90% B; 70–75 min, 90–5% B; 75–80 min, 5% B. The flow rate was maintained at 0.3 mL/min, and the injection volume was 1 μL.

Mass spectrometric detection was carried out using an electrospray ionization (ESI) source operating in both positive and negative ion modes. The operating parameters were set as follows: sheath gas (N_2_) flow rate, 30 L/min; auxiliary gas (N_2_) flow rate, 10 L/min; spray voltage, 5 kV; capillary temperature, 375 °C; capillary voltage, 60 V (positive mode) and −60 V (negative mode); tube lens voltage, 80.00 V (positive mode) and −80.00 V (negative mode). Data acquisition was performed in full scan MS/MS mode with data-dependent acquisition (DDA). The full MS resolution was set to 15,000, with a mass scan range of *m*/*z* 50 to 1500. Accurate mass measurements were applied to all mass spectral peaks, and the normalized collision energy for MS/MS fragmentation was set at 35.

#### 3.2.2. UPLC-TQS-MS/MS

Chromatographic separation was achieved on an ACQUITY UPLC I-CLASS system equipped with a Waters ACQUITY UPLC HSS T3 column (100 mm × 2.1 mm, 1.8 µm) (Waters Corporation, Milford, MA, USA). The mobile phase comprised solvent A (0.1% formic acid in water) and solvent B (acetonitrile), delivered according to the following gradient program: 0–2 min, 10% B; 2–7 min, 10–90% B; 7–8 min, 90% B; 8–8.5 min, 90–10% B; 8.5–10 min, 10% B. The flow rate was maintained at 0.3 mL/min, the column temperature was held at 30 °C, and the injection volume was 1 µL.

Mass spectrometric detection was performed using a Waters XEVO TQS system (Waters Corporation, Milford, MA, USA). Analysis was conducted in both positive and negative ionization modes with multiple reaction monitoring (MRM). Key source parameters were set as follows: capillary voltage, 3.0 kV; cone voltage, 10 V; source offset, 50 V; desolvation temperature, 500 °C; desolvation gas, 1000 L/hr; cone gas, 150 L/hr; nebulizer gas pressure, 7 bar. The retention times, precursor and product ions, and collision energies for all analytes are summarized in Table 5.

### 3.3. Solution Preparation

#### 3.3.1. Preparation of Standard Solution

Individual stock solutions were prepared by accurately weighing approximately 1 mg of each reference compound (DHC, IMP, IIMP, OPH, XAN, HSYA, CGA, FA, and LIG) and dissolving them in 70% ethanol via sonication. The concentrations of these primary stock solutions were adjusted to approximately 1 mg/mL, respectively. Subsequently, an appropriate volume of each stock solution was accurately transferred into a single 50 mL Eppendorf tube (EP tube). The mixture was then diluted with 70% ethanol to a final volume of 20 mL to obtain a mixed working standard solution, in which the mass concentration of each component was 1045, 948, 1892, 206, 56, 10,635, 2303, 2479 and 24,654 ng/mL, respectively.

#### 3.3.2. Preparation of Sample Solution

Approximately 20 mg of the sample (Batch No. 240804, 20250701, 20250702, 20250703, 20251022 and 20251023) was accurately weighed and dissolved in 10 mL of 70% ethanol. The mixture was subsequently sonicated (250 W, 40 kHz) for 30 min. After cooling to room temperature, the solution was centrifuged at 12,000 r/min for 10 min. The resulting supernatant was collected and diluted four-fold with 70% ethanol to obtain a final test solution with a concentration of 500 µg/mL.

#### 3.3.3. Method Validation

For method validation, a sample stock solution with a concentration of 5 mg·mL^−1^ was prepared by dissolving WTHXC in 70% ethanol. The impurity reference solution (1 mg·mL^−1^) was diluted to obtain a mixed impurity stock solution, in which the concentrations of DHC, IMP, IIMP, OPH, XAN, HSYA, CGA, FA and LIG were 1045, 948, 1892, 206, 56, 10,635, 2303, 2479 and 24,654 ng/mL, respectively.

A series of linearity working solutions were prepared by serially diluting the reference standard stock solution at dilution factors of 2-fold, 5-fold, 8-fold, 10-fold, 12.5-fold, 20-fold, and 50-fold. The 10-fold diluted stock solution was used for the precision test.

For reproducibility evaluation, six parallel portions of WTHXC were accurately weighed, dissolved in 70% ethanol, ultrasonicated, diluted and centrifuged. The resulting supernatants were prepared as six test solutions at approximately 500 μg/mL and injected for analysis. Method reproducibility was evaluated based on the determined contents of the nine components in the six solutions.

For stability and robustness evaluation, the sample stock solution was diluted 10-fold prior to analysis. For accuracy assessment, the sample stock solution was first diluted 5-fold, then spiked with varying volumes of the mixed impurity stock solution to attain concentration levels of 80%, 100% and 120% relative to the nine target components.

### 3.4. Transdermal Behavior Characterization

#### 3.4.1. Preparation of Skin

In this study, the skin on the back of pig ears was selected as the skin model [51,52]. All the skin tissues were purchased from the local market and collected immediately after the animals were sacrificed. Excess fat was removed using a surgical knife, and the full-thickness pig ear skin (including the stratum corneum, epidermis and dermis) was separated and prepared. The obtained skin specimens were washed three times with physiological saline, wrapped in tin foil, sealed and stored in a −20 °C refrigerator for future experiments, ensuring their availability within two weeks. The experimental samples selected were those without physical damage and abnormal skin.

#### 3.4.2. In Vitro Transdermal Permeation

In vitro transdermal permeation studies were performed using full-thickness skin from the dorsal side of pig ears. The experiment was carried out with an RT800 automatic sampling transdermal diffusion system (Raytor Instruments Co., Ltd., Shenzhen, China), equipped with a receptor chamber volume of 10 mL and an effective diffusion area of 0.785 cm^2^. The skin was fixed between the donor chamber and the receptor chamber. During the experiment, an appropriate amount of WTHXC was added to the skin for testing. The circulating water temperature of the diffusion apparatus was set at 32 °C ± 0.5 °C. The receptor chamber was filled with normal saline containing 0.05% DMSO. The medium was removed from the receptor chamber after 6 h and the content of nine components in the medium was determined using the developed UPLC- TQS-MS/MS method. As for the pig ear skin, it was immediately removed from the fixation and quickly rinsed with normal saline. After gently blotting excess water from the skin using filter paper, the skin was cut into small fragments and homogenized thoroughly. Subsequently, the skin homogenate was mixed with 2 mL of 70% ethanol (*v*/*v*), followed by ultrasonic extraction treatment. The resulting extract was centrifuged at 12,000 rpm for 10 min, and the supernatant was carefully collected. The target component content in the supernatant was determined by UPLC-TQS-MS/MS method.

## 4. Conclusions

The identification of chemical components and the development of quantitative methods are essential for ensuring the safety, stability, and quality consistency of WTHXC. In this study, UPLC-MS/MS was comprehensively employed to characterize the material basis of WTHXC and to establish corresponding quantitative methods. By correlating retention times with MS1 and MS2 data, nine components were unambiguously confirmed using authentic reference standards. Subsequently, a UPLC-MS/MS method was developed and validated for the simultaneous quantification of nine bioactive constituents in WTHXC. Through systematic optimization, baseline separation and sensitive detection were achieved within 10 min, indicating the high analytical throughput of the methods. The results of method validation revealed that the developed method is sensitive and accurate, which can be used for the quality control of the preparation. Analysis of six batches of WTHXC revealed that LIG was the most abundant constituent, while XAN was the least abundant. Furthermore, the transdermal behavior of nine components was characterized, and differences in their penetration behavior were observed, thereby providing insights into the mechanism of action of this drug from the perspective of penetration. This work represents the first reported simultaneous quantification of multi-class components in WTHXC. The quantified compounds serve as key markers with established pharmacological activities, providing a chemical basis for elucidating the efficacy material basis of the WTHXC. The developed method not only enhances the quality control system for WTHXC but also offers a practical analytical approach for multi-component quantification of similar topical herbal preparations, contributing to the modernization of traditional Chinese medicine quality standards.

## Figures and Tables

**Table 1:** Mass spectrometry information of different compounds in the WTHXC.

No.	Retention Time	Components	Formula	Theoretical *m*/*z*	Determined *m*/*z*		Deviation (ppm)	Structure
					MS1	MS2		
1	25.16	CGA (Chlorogenic acid)	C_16_H_18_O_9_	353.0873	353.0885 [M − H]^−^	135.01, 179.06, 191.02	3.40	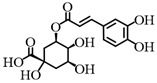
2	30.84	HSYA (Hydroxysafflor yellow A)	C_27_H_32_O_16_	611.1612	611.1596 [M − H]^−^	283.14, 325.13, 403.21	−2.62	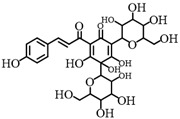
3	31.01	FA (Ferulic Acid)	C_10_H_10_O_4_	193.0501	193.0492 [M − H]^−^	134.00, 149.00, 177.96	−4.66	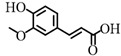
4	36.60	OPH (Oxypeucedanin hydrate)	C_16_H_16_O_6_	305.1025	305.1011 [M + H]^+^	147.03, 159.04, 175.09, 203.00	−4.59	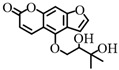
5	41.88	XAN (Xanthotoxin)	C_12_H_8_O_4_	217.0501	217.0496 [M + H]^+^	173.02, 174.05, 185.00, 201.97	−2.30	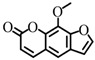
6	51.03	DHC (Dihydrocapsaicin)	C_18_H_29_NO_3_	308.2225	308.2211 [M + H]^+^	136.94, 184.09	−4.54	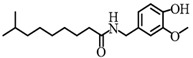
7	51.17	IMP (Imperatorin)	C_16_H_14_O_4_	271.0970	271.0959 [M + H]^+^	215.03, 202.99, 175.03, 159.03	−4.06	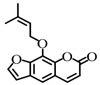
8	51.78	LIG (Ligustilide)	C_12_H_15_O_2_	191.1072	191.1062 [M + H]^+^	172.99, 145.02, 104.96	−5.23	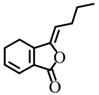
9	53.87	IIMP (Isoimperatorin)	C_16_H_14_O_4_	271.0970	271.0957 [M+H]^+^	202.99, 229.07, 175.03, 159.03	−4.8	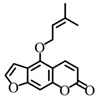

**Table 2:** The results of methodological validation.

Analytes	Calibration Curve	R^2^	Range	Precision	Repeatability	Recovery	Solution Stability	Robustness
	(y = ax + b)		(ng/mL)	(RSD, n = 6)	(RSD, n = 6)	(Mean ± RSD, n = 9)	(RSD, n = 6)	(RSD, n = 9)
DHC	y = 100.56x + 6.4441	0.9998	20.90~1045	1.25%	3.58%	101.04% ± 1.03%	5.45%	2.15%
OPH	y = 184.92x + 24.976	0.9995	41.20~2060	0.81%	4.23%	100.27% ± 1.08%	4.76%	1.14%
IMP	y = 90.474x + 5.1164	0.9999	18.96~948	0.86%	3.74%	99.83% ± 1.56%	4.52%	8.79%
IIMP	y = 189.84x + 10.609	0.9998	37.84~1892	1.45%	3.81%	100.28% ± 0.25%	4.34%	6.91%
XAN	y = 5.5796x − 0.014	0.9999	1.12~56	5.62%	2.36%	99.54% ± 1.58%	5.01%	6.77%
HSYA	y = 1229.7x − 175.23	0.9991	212.70~10,635	7.11%	9.15%	100.22% ± 1.83%	4.16%	7.11%
CGA	y = 256.6x − 21.363	0.9999	46.06~2303	2.25%	5.27%	96.51% ± 1.61%	4.18%	8.10%
FA	y = 228.34x + 12.898	0.9996	49.58~2479	6.39%	8.64%	99.43% ± 1.10%	3.73%	2.30%
LIG	y = 2222x + 238.6	0.9997	493.08~24,654	0.57%	4.29%	97.90% ± 2.72%	5.13%	0.91%

**Table 3:** Content determination of nine components in six batches of WTHXC.

Analytes	Content/(mg·g^−1^)							
Batch No.	20240804	20250701	20250702	20250703	20251022	20251023	Average	Proportion
DHC	0.209	0.242	0.2764	0.2502	0.2675	0.2776	0.2538	0.0254%
OPH	0.412	0.3066	0.3501	0.3169	0.3388	0.3516	0.3460	0.0346%
IMP	0.1896	0.7561	0.8271	0.6815	0.8015	0.8063	0.6770	0.0677%
IIMP	0.3784	0.906	0.9207	0.8836	0.9659	1.0206	0.8459	0.0846%
XAN	0.0112	0.0253	0.0322	0.0291	0.0272	0.0298	0.0258	0.0026%
HSYA	2.127	1.8711	2.9076	2.7003	2.0761	2.9026	2.4308	0.2431%
CGA	0.4606	0.0897	0.1317	0.1141	0.1039	0.1433	0.1739	0.0174%
FA	0.4958	0.4755	0.6073	0.5150	0.6078	0.5969	0.5497	0.0550%
LIG	4.9308	5.2018	6.4937	6.2966	6.3919	6.7791	6.0157	0.6016%

**Table 4:** In vitro transdermal permeation and intradermal retention of nine components in WTHXC across three porcine skin samples.

Analytes	In Vitro Retention in Skin (μg·cm^−2^)				In Vitro Transdermal Permeation (μg·cm^−2^)			
No.	Porcine Skin1	Porcine Skin2	Porcine Skin3	Average Value	Porcine Skin1	Porcine Skin2	Porcine Skin3	Average Value
DHC	0.6128	0.1452	0.3243	0.3608	0.0000	0.0001	0.0001	0.0001
OPH	9.0247	9.2013	10.8628	9.6963	0.2480	1.5546	4.5372	2.1133
IMP	4.9714	0.7362	1.5027	2.4035	0.0005	0.0100	0.0353	0.0153
IIMP	2.5282	0.3980	0.8174	1.2478	0.0001	0.0004	0.0016	0.0007
XAN	0.6064	0.2281	0.2473	0.3606	0.0009	0.0367	0.0312	0.0229
HSYA	44.8872	18.6640	12.9593	25.5035	1.1806	13.2195	80.2765	31.5589
CGA	2.4210	0.8359	0.7681	1.3417	0.0539	0.6425	2.0615	0.9193
FA	15.4290	6.3153	5.0445	8.9296	0.1016	0.1103	0.2613	0.1577
LIG	36.2575	3.5644	7.1073	15.6431	0.0002	0.2658	0.2188	0.1616

**Table 5:** MRM parameters of 9 components in WTHXC.

NO.	Analytes	RT (min)	*m*/*z*		Collision Energy (v)
			MS1	MS2	
1	HSYA	3.64	611.4 [M − H]^−^	491.2	23
2	CGA	3.70	353.2 [M − H]^−^	191.2	18
3	FA	4.61	193.2 [M − H]^−^	134.2	18
4	OPH	5.23	305.2 [M + H]^+^	203.3	21
5	XAN	5.91	217.1 [M + H]^+^	202.2	20
6	DHC	6.95	308.1 [M + H]^+^	137.6	14
7	IMP	7.02	271.3 [M + H]^+^	203.2	12
8	LIG	7.24	191.2 [M + H]^+^	173.3	16
9	IIMP	7.36	271.3 [M+H]^+^	203.0	12

## Data Availability

The original contributions presented in this study are included in the article. Further inquiries can be directed to the corresponding authors.

## Abbreviations

The following abbreviations are used in this manuscript:

WTHXC	WenTong HuoXue Cream
MRM	Multiple reaction monitoring
DPN	Diabetic peripheral neuropathy
RSD	Relative standard deviation
UPLC-MS/MS	Ultra Performance Liquid Chromatography-Tandem Mass Spectrometry
DHC	Dihydrocapsaicin
OPH	Oxypeucedanin hydrate
IMP	Imperatorin
IIMP	Isoimperatorin
XAN	Xanthotoxin
HSYA	Hydroxysafflor yellow A
CGA	Chlorogenic acid
FA	Ferulic Acid
LIG	Ligustilide
